# A Highly Sensitive Electrochemical Glucose Sensor Based on Room Temperature Exfoliated Graphite-Derived Film Decorated with Dendritic Copper

**DOI:** 10.3390/ma14175067

**Published:** 2021-09-04

**Authors:** Jiaxin Tang, Luo Wei, Shuaijie He, Jihui Li, Ding Nan, Liqiang Ma, Wanci Shen, Feiyu Kang, Ruitao Lv, Zhenghong Huang

**Affiliations:** 1 State Key Laboratory of New Ceramics and Fine Processing, School of Materials Science and Engineering, Tsinghua University, Beijing 100084, China; jiaxintang1008@163.com (J.T.); 18813136963@163.com (L.W.); heshuaijie@cug.edu.cn (S.H.); shenwc@mail.tsinghua.edu.cn (W.S.); fykang@tsinghua.edu.cn (F.K.); lvruitao@tsinghua.edu.cn (R.L.); 2 School of Chemical & Environmental Engineering, China University of Mining & Technology (Beijing), Beijing 100083, China; mlqiang@cumtb.edu.cn; 3 College of Chemistry and Chemical Engineering, Inner Mongolia University, West University Street 235, Hohhot 010021, China; nan1980732@163.com; 4 Inner Mongolia Key Laboratory of Graphite and Graphene for Energy Storage and Coating, Aimin Street 49, Hohhot 010051, China; 5 Key Laboratory of Advanced Materials (MOE), School of Materials Science and Engineering, Tsinghua University, Beijing 100084, China

**Keywords:** exfoliated graphite-derived film, dendritic Cu structures, enzyme-free glucose sensor

## Abstract

An ultrasensitive enzyme-free glucose sensor was facilely prepared by electrodepositing three-dimensional dendritic Cu on a room temperature exfoliated graphite-derived film (RTEG-F). An excellent electrocatalytic performance was demonstrated for glucose by using Cu/RTEG-F as an electrode. In terms of the high conductivity of RTEG-F and the good catalytic activity of the dendritic Cu structures, the sensor demonstrates high sensitivities of 23.237 mA/mM/cm^2^, R^2^ = 0.990, and 10.098 mA/mM/cm^2^, R^2^ = 0.999, corresponding to the concentration of glucose ranging from 0.025 mM to 1.0 mM and 1.0 mM to 2.7 mM, respectively, and the detection limit is 0.68 μM. In addition, the Cu/RTEG-F electrode demonstrates excellent anti-interference to interfering species and a high stability. Our work provides a new idea for the preparation of high-performance electrochemical enzyme-free glucose sensor.

## 1. Introduction

In the process of preventing diabetes, it is very important to obtain the blood glucose concentration quickly and effectively [1]. Therefore, a variety of principles and methods have been explored by researchers to develop a new type of fast, stable, reliable, cheap, and efficient glucose sensor [2]. Enzyme glucose sensors are very popular and have good selectivity and sensitivity, which is mainly due to the unique selectivity of glucose oxidase (GOx) to glucose [3]. Enzyme-free glucose sensors have also received more attention recently, and they also need catalytic active sites to react with glucose, which is similar to enzyme glucose sensors [4]. The enzyme-free glucose sensors avoid the shortcomings of enzyme glucose sensors, one of which is being affected easily by environmental factors (temperature, humidity, and toxic chemicals) [5]. The nonenzymatic sensors also have high usefulness in body fluids (blood/urine) and food samples’ glucose detection [6].

It is essential for a high sensitivity enzyme-free glucose sensor to use the active substances with good electrocatalytic activity. Metal nanomaterials such as Cu [7,8], Ni [9], Au [10], and Pt [11]; metal oxides such as CuO [12], Co_3_O_4_ [13], and MnO_2_ [14]; and bimetallic nanoparticles such as Cu-Ni [6] and Pt-Pd [15], were widely applied in non-enzyme glucose sensors filed based on their high electrocatalytic performance and less affected by the environment. Generally, the electrochemical catalysts were loaded on the support to catalyze the glucose. The support material with a high conductivity and a high specific surface area can make the whole sensor exhibit excellent sensitivity after uniformly loading of active materials. Up to now, graphene [16], carbon nanotubes [17], ITO conductive films [9], etc., were widely studied as carrier materials. However, most methods of preparing and fabricating the support were complex and the active material fell off easily, which reduced the reproducibility or stability of detection [18]. In order to overcome the disadvantages of this electrode, self-supporting electrodes were prepared with simple method and one-step electrodeposition metal or metal oxide nanoparticles directly on support materials [19].

The conjugated polymers (CPs)-based materials have a low detection limit and a high sensitivity when used as biosensors owing to the unique photoelectric characteristics [20]. The CPs have been widely studied as substrate materials in enzyme-free [21] and enzymatic [22] glucose sensors. However, compared with flexible graphite sheets substrate, the preparation process of conjugated polymer is more complex. Flexible graphite sheets [23] are usually made of exfoliated graphite and remain traces of graphite flake pressing on the sheets surface. The preparation method of exfoliated graphite is very simple. The high electrical conductivity and well flexibility make the flexible graphite sheets used in many fields, and these advantages are also suitable for use as self-supporting substrate material for sensors, which can also exploit another application field of flexible graphite sheet.

In this work, the support material of the sensor was obtained by a simple pressing method using our lab-made room temperature exfoliated graphite-derived film (RTEG-F) [24], and the dendritic copper structures were uniformly loaded on the surface of substrate by one-step electrodeposition to prepare a highly efficient glucose sensor.

## 2. Materials and Methods

### 2.1. Materials

Natural graphite flakes, sulfuric acid (H_2_SO_4_) and hydrogen peroxide (H_2_O_2_) were purchased from Aladdin, Reagent Co., Ltd. (Shanghai, China). Potassium chloride (KCl), copper sulfate pentahydrate (CuSO_4_·5H_2_O), glucose, uric acid (UA), dopamine hydrochloride (DA), sodium chloride (NaCl), acetaminophenol (AP), L-ascorbic acid (AA), and sodium hydroxide (NaOH) were obtained from Sinopharm Chemical Reagent Co., Ltd. (Shanghai, China). mannitol, lactose, and maltose were purchased from Macklin Biochemical Technology Co., Ltd. (Shanghai, China).

The apparatus involves scanning electron microscopy (SEM, LEO1530) (Carl Zeiss Microscopy GmbH, Oberkochen, Germany) for characterizing the microstructures of RTEG-F and Cu/RTEG-F, X-ray Diffraction (XRD, Bruker D8 ADVANCE) (Bruker AXS GmbH, Karlsruhe, Germany) for studying the material composition. Electrochemical deposition, amperometric measurements and cyclic voltammetry (CV) were performed in VSP-300 electrochemical interface (BioLogic China, Beijing, China).

### 2.2. Methods

#### 2.2.1. Preparation of RTEG-F

Firstly, room temperature exfoliated graphite (RTEG) was obtained by our previously reported room temperature exfoliation method [24]. Briefly, graphite powder (0.5 g) was added into 7 mL of H_2_SO_4_ solution with the ice-water bath temperature below 5 °C, then 1.8 mL of H_2_O_2_ were slowly dripped into the continuously stirred mixture. After 0.5 h of intercalation at 5 °C, the mixture samples were transferred to drying oven with a temperature of 30 °C and held at that temperature for 4 h. The dried samples were then placed in a muffle furnace with the temperature of 350 °C for 1 h to remove excess sulfur; the obtained worm-like particles were called room temperature exfoliated graphite. Then, the RTEG-derived films (RTEG-F) were achieved by further compressing the RTEG through a roll squeezer (Hefei kejing Material Technology Co., Ltd., Hefei, China) set to 0.2 mm thick.

In comparison, we prepared high temperature exfoliated graphite-derived film (HTEG-F) by conventional method. The HTEG was synthesized by adding graphite to H_2_SO_4_ and H_2_O_2_ solution (the molar ratio of H_2_SO_4_/H_2_O_2_ was 55/1). The obtained compounds were filtered with water several times, and dried in the oven at 80 °C. The products were converted to HTEG by rapidly heating at 900 °C in muffle furnace. Finally, the HTEG-F was achieved by further compressing the HTEG through a roll squeezer.

#### 2.2.2. Preparation of Cu/RTEG-F Electrode

**Scheme 1:**
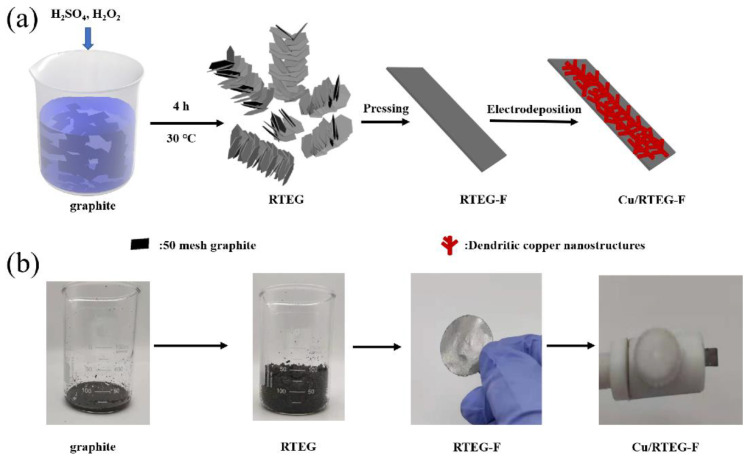
(**a**) Schematic illustration of the preparation of RTEG-F and Cu/RTEG-F samples. (**b**) Photographs of the graphite, RTEG, RTEG-F, and Cu/RTEG-F. Here, RTEG-F and Cu/RTEG-F denote room temperature exfoliated graphite-derived film and copper modified room temperature exfoliated graphite-derived film, respectively.

The Cu/RTEG-F electrodes were prepared by electrodeposition method. Simply, the prepared RTEG-F were cut into pieces (area immersed in solution was 2 mm × 3 mm) as working electrodes. Platinum wire and Ag/AgCl were used as the counter electrode and the reference electrode, respectively. The electrode position condition is −0.4 V for 8 min in the 0.01 M CuSO_4_ contains 0.1 M KCl. Afterwards, the Cu/RTEG-F was cleaned with ultrapure water. The schema of preparing RTEG-F and Cu/RTEG-F was shown in Scheme 1.

#### 2.2.3. Electrochemical Tests

The electrochemical tests were also implemented in three electrodes system (Tianjin AIDAhengsheng Science-Technology Development Co., Ltd., Tianjin, China). The alkaline electrolyte was 40 mL of 0.1 M NaOH aqueous solution, and the range of CV tests were from −1.0 V–1.0 V or 0 V–1.0 V. The detection voltage was 0.65 V. Glucose was added into NaOH aqueous solution at an interval of 50 s and stirred at a certain speed.

## 3. Results and Discussion

### 3.1. Characterization

**Figure 1:**
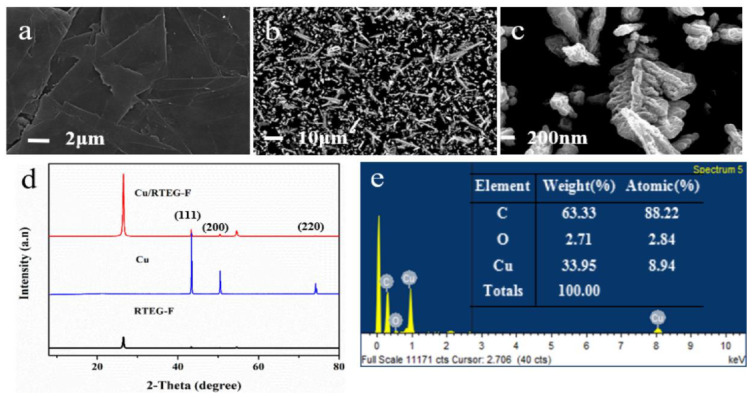
SEM images of (**a**) pure RTEG-F and (**b**,**c**) Cu/RTEG-F at a deposition time of 8 min. (**d**) XRD pattern of Cu/RTEG-F. (**e**) The EDS pattern and elemental contents of Cu/RTEG-F.

Figure 1 and Figure S1 show the microstructural morphologies of the pristine RTEG-F and Cu/RTEG-F at different electrodeposition time (6–12 min). The surface of the pristine RTEG-F was clean and homogeneous. Graphite sheets were stacked up together, and there were many creases between sheets (Figure 1a). Thus, enough attachment sites are provided for the Cu particles to be adhered to the surface of the RTEG-F. When electrodeposited for 6 min, a large amount of Cu nanoparticles deposited on RTEG-F surface, and the nanoparticles are circular and the diameter are about 100 nm (Figure S1a,b). After 8 min of electrodeposition, the surface of RTEG-F attached more Cu nanoparticles and dendritic copper structures began to form (Figure 1b). The dendritic Cu structures can be seen more clearly in high-resolution SEM image in Figure 1c. It is well known that the three-dimensional dendritic structures have a high specific surface area and can enable rapid transport of electrolyte [25]. Therefore, 3D Cu dendritic structures are effective catalyst materials for glucose. When the deposition time was extended to 10 min, relatively complete dendritic structures were formed as shown in Figure S1c,d. It can be found from the comparison between Figure 1c and Figure S1d that the size of the dendritic structures formed after 10 min of deposition is about 5 times larger than that formed after 8 min of deposition. When the electrodeposition time increased to 12 min, the dendrites grew bigger and more uniform on the electrode surface as shown in Figure S1e,f. The phenomenon is similar to the reported literature [26].

To investigate the Cu structures deposited on the RTEG-F surface, we carried out XRD test for pristine RTEG-F, copper powder, and Cu/RTEG-F samples (Figure 1d). The RTEG-F sample displayed two peaks at 2θ = 26.26° and 54.7°, which corresponds to the typical characteristic diffraction peak of graphite material, indicating that the RTEG-F still maintains a complete graphite structure after rolling, and it has the characteristics of high conductivity of graphite materials. The Cu/RTEG-F has three diffraction peaks except those possessed by RTEG-F at 2θ = 43.34°, 50.4° and 73.6°, corresponding to Miller index (111), (200) and (220) of copper face centered cubic lattice, which indicates that copper was successfully deposited on the surface of RTEG-F. From the EDS spectra (Figure 1e), the atom ratio of C/O/Cu is 63.33: 2.71: 33.95, which further proved the copper deposition.

### 3.2. Electrochemical Behavior of Cu/RTEG-F Electrodes

**Figure 2:**
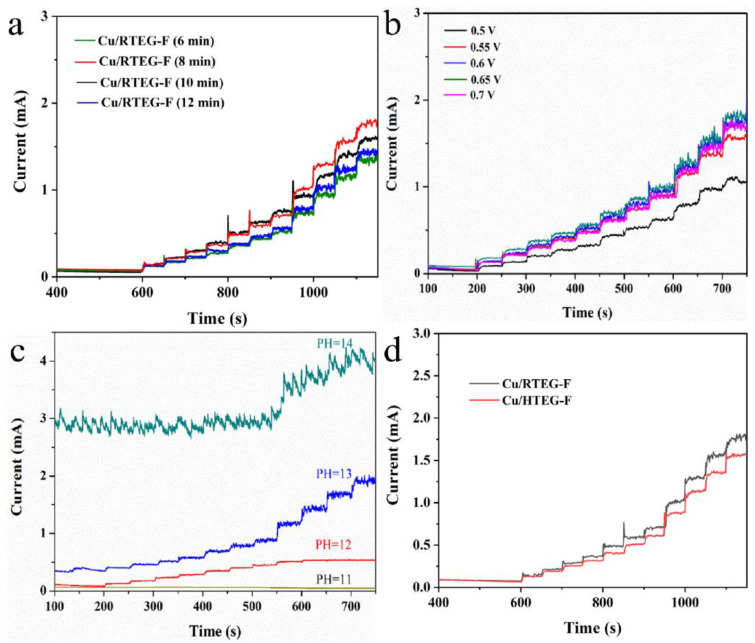
(**a**) Amperometric responses of Cu/RTEG-F electrodes prepared at four electrodeposition times (6 min, 8 min, 10 min, 12 min) with successive additions of glucose into 0.1 M NaOH every 50 s at 0.65 V. (**b**) Amperometric responses of Cu/RTEG-F electrodes deposited for 8 min with continuous infusion of glucose in 0.1 M NaOH at various potentials (0.5–0.7 V). (**c**) Amperometric responses of Cu/RTEG-F electrodes deposited for 8 min with continuous infusion of glucose at various pH from 11 to 14. (**d**) Amperometric responses of Cu/RTEG-F electrode and Cu/RTEG-F electrode deposited for 8 min with continuous infusion of glucose into 0.1 M NaOH every 50 s at 0.65 V.

We investigated the influence factors of the preparation and detection of the prepared electrode, including deposition time, detection potential and pH value of electrolyte as shown in Figure 2a–c. Figure 2a shows amperometric responses of Cu/RTEG-F electrode prepared at electrodeposition time of 6 min, 8 min and 12 min with successive additions of glucose into NaOH solution every 50 s. We tested the sensitivity of the sensor under different electrodeposition time. The detection range of glucose was 1.0 to 2.6 mM, the sensitivities after 6, 8, 10 and 12 min of deposition were 8.081 mA/mM/cm^2^, 10.506 mA/mM/cm^2^, 9.512 mA/mM/cm^2^, and 8.470 mA/mM/cm^2^, respectively. The best corresponding current appears at 8 min of copper deposition owing to the ‘optimization’ of nanocluster dimension and nanocluster loading [27]. When deposited for 8 min, there are smaller Cu nanostructures and bigger surface area of the nanostructures and thus a higher electrochemical response. On the other hand, the electrode deposited for 6 min shows smaller electrochemical response owing to a lower amount of copper nanocluster (electrode loading) on the surface of the electrode. Thus, the electrode deposited for 8 min shows the best compromises between nanocluster loading and nanocluster dimension, and the sample with copper deposition time of 8 min was employed at all tests. Then, the working potential (0.5–0.7 V) also was explored by chronoamperometry in Figure 2b. We tested the sensitivity of the sensor under different potential. The detection range of glucose was from 1.0 mM to 2.6 mM, the sensitivities at 0.5 V, 0.55 V, 0.6 V, 0.65 V, and 0.7 V were 8.081 mA/mM/cm^2^, 9.241 mA/mM/cm^2^, 10.269 mA/mM/cm^2^, 10.685 mA/mM/cm^2^, and 10.067 mA/mM/cm^2^, respectively. The highest sensitivity occurs when the voltage is 0.65 V. The pH value of alkaline solution also has a great influence on the detection results. Therefore, we tested the catalytic performance of the modified Cu/RTEG-F electrode for glucose at different pH values (Figure 2c). There was no significant response in the low pH, such as 11. The current response gradually increases with the pH from 12 to 14, but the current response become instability at the pH value of 14. The current response gradually increases with the pH from 12 to 13 because the glucose was more easily oxidized at higher pH and more hydroxyl radical was formed. However, when the pH was 14, the current response was lower owing to the limited loading of copper on the electrode [28,29]. The best current response was obtained at pH = 13, corresponding to 0.1 M of NaOH, which was used in all experiments.

We also compared the response of copper deposited on RTEG-F and HTEG-F substrates towards glucose as shown in Figure 2d. We tested the sensitivity of Cu/RTEG-F and Cu/RTEG-F electrodes. The detection range of glucose was from 1.0 mM to 2.6 mM. The sensitivity of Cu/RTEG-F was 10.506 mA/mM/cm^2^, and the sensitivity of Cu/HTEG-F was 9.660 mA/mM/cm^2^. The Cu/RTEG-F has higher sensitivity than Cu/RTEG-F electrode, which is attributed to the high electrical conductivity of RTEG-F. As our team reported previously, the electrical resistivity of HTEG-F is higher than RTEG-F with the same density. Under the same test conditions, Cu/RTEG-F has better current response than Cu/HTEG-F, so RFGF was selected as the substrate material.

**Figure 3:**
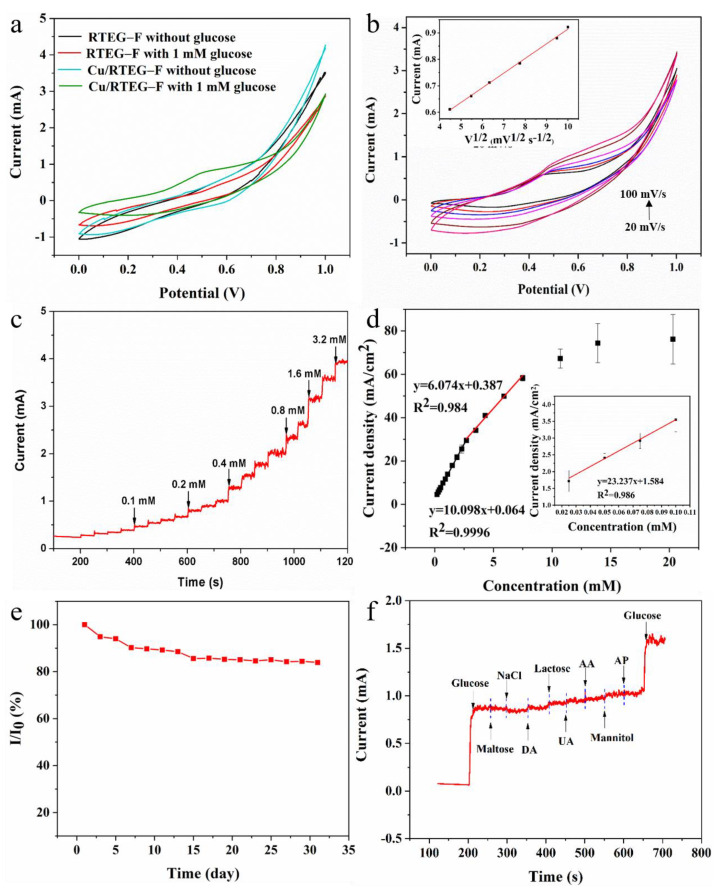
(**a**) CV curves of pristine RTEG-F and Cu/RTEG-F electrodes with or without the addition of 1.0 mM glucose at a scan rate of 50 mV s^−1^. (**b**) CV curves of Cu/RTEG-F electrode at different scanning rates in NaOH solution containing 1.0 mM glucose. Inset: dependence of the glucose oxidation peak current on the scanning rate. (**c**) Amperometric current responses of Cu/RTEG-F electrode continuous adding of various concentration of glucose (e.g., adding four times of 1 μL of 1.0 M glucose solution into NaOH solution will increase the concentration of glucose in the system to 0.025 mM every time). (**d**) The relationship between the glucose concentration (2.7 mM to 20.3 mM) and the corresponding current density. Inset: the relationship between the glucose concentration (0.025 mM to 2.7 mM) and the corresponding current density (*n* = 3). (**e**) The relationship between the glucose concentration and the corresponding current density. (**f**) The current response of the Cu/RTEG-F electrode by the addition of 1.0 mM glucose, 0.1 mM maltose, NaCl, DA, lactose, UA, AA, mannitol, AP, and 1.0 mM glucose into 0.1 M NaOH solution.

The electrochemical performance of pristine RTEG-F and Cu/RTEG-F electrodes were detected by CV test in 0.1 M NaOH solutions under existence or nonexistence of 1.0 mM glucose with potential of 0–1.0 V (Figure 3a). There was not obvious oxidation peak with or without glucose, indicating that the pristine RTEG-F could not be used for detecting glucose. There was also no oxidation peak for Cu/RTEG-F electrode without glucose. However, an anodic oxidation peak can be apparently observed with the existence of glucose at the potential of 0.52 V. It suggests that the copper is an active substance that reacts with glucose. Figure S2 shows that Cu can be oxidized to Cu (I), Cu (II), and Cu (III) in alkaline environment. The widely accepted catalytic mechanism of copper for glucose is that Cu (III) is a strong oxidant and the glucose can be oxidized into gluconate [30].

Figure 3b shows the CV curves of Cu/RTEG-F electrode at different scanning rates in NaOH solution containing 1.0 mM glucose. The oxidation peak appears at the potential of 0.52 V and the peak current increases with the increase of sweep speed. The inset curve shows that the peak current (I) was proportional to the square root of scanning rate (V^1/2^), illustrating that the reaction of glucose at the Cu/RTEG-F electrode is a diffusion-controlled route [31]. Figure 3c shows the amperometric current response of glucose at various concentrations by putting into 0.1 M NaOH. The Cu/RTEG-F electrode reacted rapidly and reached a stable current plateau. The lines in Figure 3d show the relationship between the concentration and the current of glucose. The sensor demonstrates a high sensitivity of 23.237 mA/mM/cm^2^, R^2^ = 0.990 and 10.098 mA/mM/cm^2^, R^2^ = 0.999 corresponding to the concentration of glucose ranging from 0.025 mM to 1.0 mM and 1.0 mM to 2.7 mM, also has a sensitivity of 6.074 mA/mM/cm^2^, R^2^ = 0.984, corresponding to 2.7 mM to 7.5 mM, respectively, and the detection limit was about 0.68 μM (signal/noise = 3). Table 1 lists the Cu/RTEG-F glucose detection results in comparison with those of other copper based non enzyme sensors, revealing that the sensor in this study has higher sensitivity. Table 2 lists several different types of glucose sensors, including optical glucose sensor, liquid chromatography glucose sensor, Raman glucose sensor, electrochemical enzyme glucose sensor, and our electrochemical non-enzyme glucose sensor, showing that the sensor has good performance.

The high sensitivity of the present sensor mainly depends on the following two factors. On one hand, the high conductivity of RTEG-F is helpful for the electron transfer, and the stacked graphite sheets provide more adsorption sites for the deposition of catalytic active materials. On the other hand, based on the ‘optimization’ of nanocluster dimension and nanocluster loading, the dendritic copper deposited has a high specific surface area and therefore has a high current response. Based on the above reasons, the Cu/RTEG-F achieves the largest current response.

### 3.3. Stability, Anti-Interference and Reproducibility of Cu/RTEG-F Electrode

Figure 3e shows the percentage of current response to the original current after different days of storage. The electrochemical property of Cu/RTEG-F sensor remained above 83% after a month, indicating good stability. Considering many interfering factors in blood glucose detection, the anti-interference performance was further investigated by amperometric responses with successively adding 1.0 mM glucose, 0.1 mM maltose, NaCl, DA, lactose, UA, AA, mannitol, AP, and 1.0 mM glucose into 0.1 M NaOH solution. Figure 3f shows that the response currents are rapidly produced once adding glucose. However, we cannot observe obvious current response when adding other types of interfering material, indicating that Cu/RTEG-F sensor exhibits a good anti-interference ability. The electrode was also tested using interference species at concentration 10 times higher than the analyte one as shown in Figure S3a,b. Compared with glucose, the generated current is smaller. This test further proves that the sensor has good anti-interference ability. Five electrodes were used to test the reproducibility of Cu/RTEG-F that prepared by using the same procedure. The relative standard deviation (RSD) of five sensors was 5.5% (Figure S4), indicating the sensor has a good reproducibility. The electrode repeatability was also tested with five times, the RSD was 3.7%, indicating the sensor has a good repeatability.

### 3.4. Glucose Detection in Real Human Serum Sample

The utilization of Cu/RTEG-F sensor was explored in human serum according to previous reports [2,32]. and the recovery of glucose was calculated by adding glucose to the solution containing human serum [40]. A total of 0.1ml of human serum was injected into 9.9 mL of 0.1 M NaOH solution with stirring and the concentration of the tested serum was calculated through the generated current and the sensitivity of the sensor. The results are listed in Table 3, indicating that the developed sensor has strong potential in real application.

## 4. Conclusions

In summary, we present a highly sensitive electrochemical glucose sensor by electrodepositing dendritic copper structures onto room temperature exfoliated graphite-derived film. The sensor demonstrates a wide linear range and a high sensitivity owing to the synergetic effect of excellent catalytic activity, large reaction surface area of three-dimensional dendritic Cu and high conductivity of RTEG-F. We have developed a simple preparation process of the glucose sensor with a high-efficiency detection performance. It opens a new field for the application of exfoliated graphite and also provides a new insight for the preparation of sensitive sensor substrate.

## Figures and Tables

**Table 1:** Comparison with other copper based non enzyme glucose sensors.

Electrode Material	Liner Range (mM)	Detection Limit (μM)	Sensitivity (mA/mM/cm^2^)	Reference
Cu NPs@NiF	0.002–0.65 and 0.65–6.0	0.5	2.679 and 1.122	[19]
Cu NPs/rGO	0.01–1.2	3.4	0.448	[32]
Cu NWAs @Ti/Cr/Si	0.002–2.156	1.87	1.067	[33]
Cu nanoparticles @MWCNTs	0.5–7.5	2.0	0.922	[34]
Cu@C	0.001–0.06	1.0	1.20	[35]
Cu@MWCNTs	0–7.5	1.0	1.096	[36]
Cu-PAni/ITO	0.02–1.0	5.0	4.140	[7]
Cu/RTEG-F	0.025–1.0, 1.0–2.7	0.68	23.237 and 10.098	This work

Note: The “@” means “loaded”.

**Table 2:** Comparison of different types of glucose sensors.

Types	Liner Range	Detection Limit	Sensitivity	Reference
Optical glucose sensor	0–0.5 mg/mL	Not reported	85.4 mg/mL	[37]
Liquid chromatography glucose sensor	1.1–113.9 μM	1.1 μM	Not reported	[38]
Raman glucose sensor	0.5–5.0 mM	0.005 mM	Not reported	[39]
electrochemical enzyme glucose sensor	0–5.0 mM	0.05 mM	288.86 μA/mM/cm^2^	[3]
electrochemical non-enzyme glucose sensor	0.025–1.0 mM, 1.0–2.7 mM	0.68 μM	23.237 mA/mM/cm^2^ and 10.098 mA/mM/cm^2^	This work

**Table 3:** Results for determination of glucose in human blood serum (*n* = 3).

Sample	Handheld Glucose Meter (mM)	Our Sensor (mM)	RSD (%)	Added (mM)	Recovery (%)
1	4.8	4.86	3.53	1.0	95.2
2	4.3	4.31	3.93	1.0	96.7

## Data Availability

Data are available on demand.

## Supplementary Materials

The following are available online at https://www.mdpi.com/article/10.3390/ma14175067/s1, Figure S1, SEM images of Cu/RTEG-F at different deposition time: 6 min (a,b), 10 min (c,d), 12 min (e,f); Figure S2, CV curves of Cu/RTEG-F electrode existence (red line) or nonexistence (black line) glucose in 0.1 M NaOH solution with potential −1.0 V to 1.0 V; Figure S3, (a) The current response of the Cu/RTEG-F electrode by the addition of 0.5 mM glucose, 5.0 mM NaCl, AP, UA, DA and Lactose into 0.1 M NaOH solution. (b) Increased current value after adding different interferents; Figure S4, (a) The reproducibility of Cu/RTEG-F electrode, (b) The repeatability of Cu/RTEG-F electrode.
