# Supplementary material for: A Highly Sensitive Electrochemical Glucose Sensor Based on Room Temperature Exfoliated Graphite-Derived Film Decorated with Dendritic Copper
Source: Materials (Basel). 2021 Sep 4;14(17):5067. doi: 10.3390/ma14175067 (PMC8433722; doi:10.3390/ma14175067)
Supplement: Supplementary file 1 [file materials-14-05067-s001.zip › materials-1311842-supplementary.pdf]

materials

Supplementary Materials

A Highly Sensitive Electrochemical Glucose Sensor Based on Room Temperature Exfoliated Graphite-Derived Film Decorated with Dendritic Copper

Jiaxin Tang [1,2,†], Luo Wei [1,2,†], Shuaijie He [1], Jihui Li [2,*], Ding Nan [3,4], Liqiang Ma [2], Wanci Shen [1], Feiyu Kang [1], Ruitao Lv [1,5] and Zhenghong Huang [1,5,*]

1 State Key Laboratory of New Ceramics and Fine Processing, School of Materials Science and Engineering, Tsinghua University, Beijing 100084, China; jiaxintang1008@163.com (J.T.); 18813136963@163.com (L.W.); heshuaijie@cug.edu.cn (S.H.); shenwc@mail.tsinghua.edu.cn (W.S.); fykang@tsinghua.edu.cn (F.K.); lvruitao@tsinghua.edu.cn (R.L.)
2 School of Chemical & Environmental Engineering, China University of Mining & Technology (Beijing), Beijing 100083, China; mlqiang@cumtb.edu.cn
3 College of Chemistry and Chemical Engineering, Inner Mongolia University, West University Street 235, Hohhot 010021, China; nan1980732@163.com
4 Inner Mongolia Key Laboratory of Graphite and Graphene for Energy Storage and Coating, Aimin Street 49, Hohhot 010051, China
5 Key Laboratory of Advanced Materials (MOE), School of Materials Science and Engineering, Tsinghua University, Beijing 100084, China
* Correspondence: lijihui@cumtb.edu.cn (J.L.); zhhuang@tsinghua.edu.cn (Z.H.)
† These authors contributed equally to this work.

Citation: Tang, J.; Wei, L.; He, S.; Li, J.-H.; Nan, D.; Ma, L.; Shen, W.; Kang, F.; Lv, R.; Huang, Z.-H. A Highly Sensitive Electrochemical Glucose Sensor Based on Room Temperature Exfoliated Graphite-Derived Film Decorated with Dendritic Copper. *Materials* **2021**, *14*, 5067. https://doi.org/10.3390/ma14175067

Academic Editor: Enrico Negro

Received: 8 July 2021
Accepted: 2 September 2021
Published: 4 September 2021

Publisher's Note: MDPI stays neutral with regard to jurisdictional claims in published maps and institutional affiliations.

Copyright: © 2021 by the authors. Licensee MDPI, Basel, Switzerland. This article is an open access article distributed under the terms and conditions of the Creative Commons Attribution (CC BY) license (http://creativecommons.org/licenses/by/4.0/).

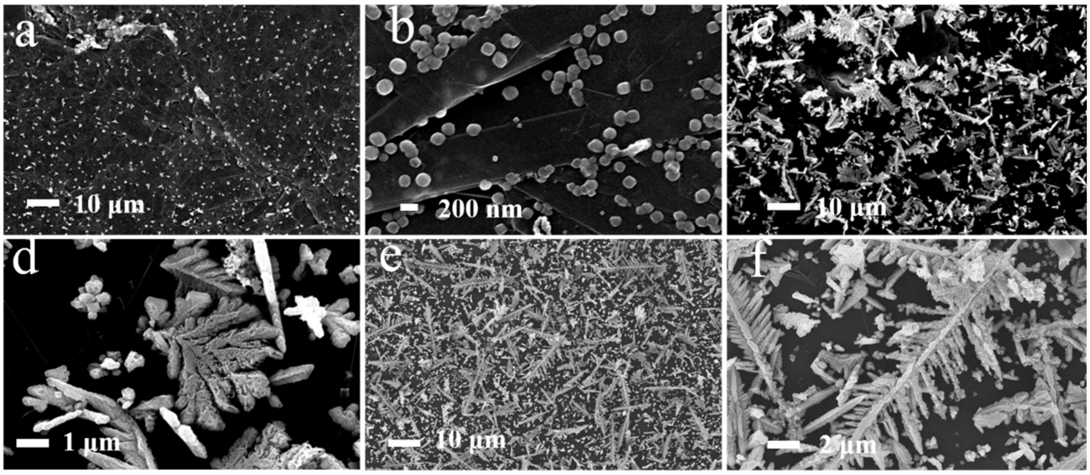

Figure S1. SEM images of Cu/RTEG-F at different deposition time: 6 min (**a**,**b**), 10 min (**c**,**d**), 12 min (**e**,**f**).

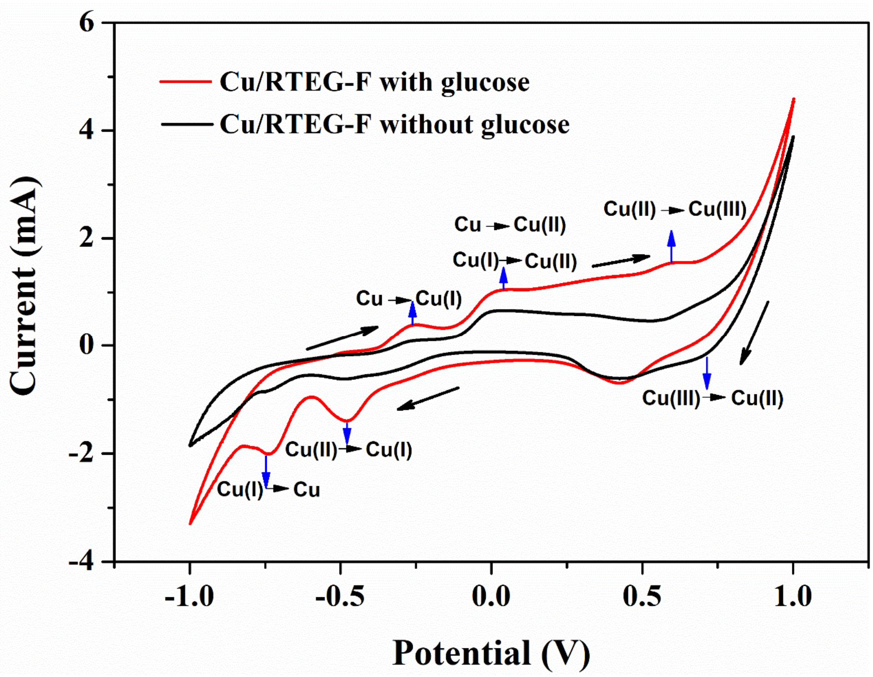

Figure S2. CV curves of Cu/RTEG-F electrode existence (red line) or nonexistence (black line) glucose in 0.1 M NaOH solution with potential -1.0 V to 1.0 V.

Figure S2 shows CV curves of Cu/RTEG-F electrodes existence (red line) or nonexistence (black line) glucose in 0.1 M NaOH solution at potential from −1.0 V to 1.0 V. According to previous reports[1–4], Cu on RTEG-F substrate was oxidized to Cu (Ⅰ)，such as CuOH at about −0.3 V.

$$Cu + OH^{-} \rightarrow CuOH + e^{-} \quad (1)$$

Cu or Cu (Ⅰ) was oxidized to Cu (II) (Cu $(OH)_2$ or CuO) duo to the exist of OH^- at about –0.15 V.

$$Cu+2OH^{-} \rightarrow Cu\,(OH)_2+2e^{-} \quad (2)$$

$$Cu + 2OH^{-} \rightarrow CuO + H_2O + 2e^{-} \quad (3)$$

$$CuOH + 2OH^{-} \rightarrow Cu\ (OH)_2 + 2e^{-} \quad (4)$$

The exact mechanism of glucose catalyzed and oxidized by copper- based electrode in alkaline medium is not clear. The most widely accepted mechanism is Cu (II) was further oxidized to Cu (III) such as CuOOH, and Cu (III) acted as an electronic medium to catalyze glucose at about 0.6 V–0.8 V.

$$CuO + OH^{-} \rightarrow CuOOH + e^{-} \quad (5)$$

$$Cu\ (OH)_2 + OH- \rightarrow CuOOH + H_2O + e^{-} \quad (6)$$

Glucose was catalyzed by Cu (III) become glucoselactone, while the Cu (III) was reduced to Cu (II) at about 0.55 V.

$$Cu\ (III) + glucose \rightarrow glucoselactone + Cu\ (II) \quad (7)$$

The reduction peaks at −0.5 V and −0.75 V of the reverse scanning correspond to Cu (II) →Cu (I) and Cu (I) → Cu, respectively. In addition, in the presence of glucose, there is no peak of Cu (III) →Cu (II) conversion, indicating that Cu (III) catalyzes the oxidation of glucose to gluconic acid, which is consumed at the same time. This phenomenon also proves that the oxidation of glucose by copper- based glucose sensor is irreversible.

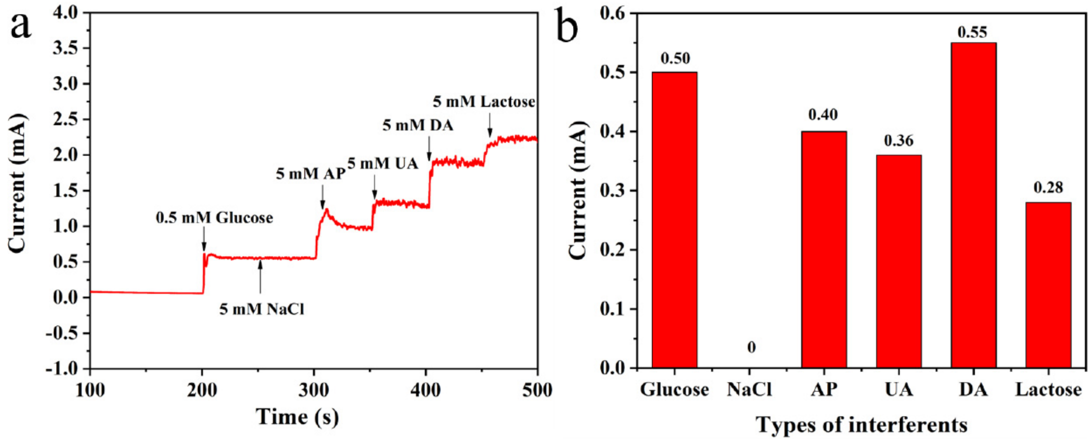

Figure S3. (**a**) The current response of the Cu/RTEG-F electrode by the addition of 0.5 mM glucose, 5.0 mM NaCl, AP, UA, DA and Lactose into 0.1 M NaOH solution. (**b**) Increased current value after adding different interferents.

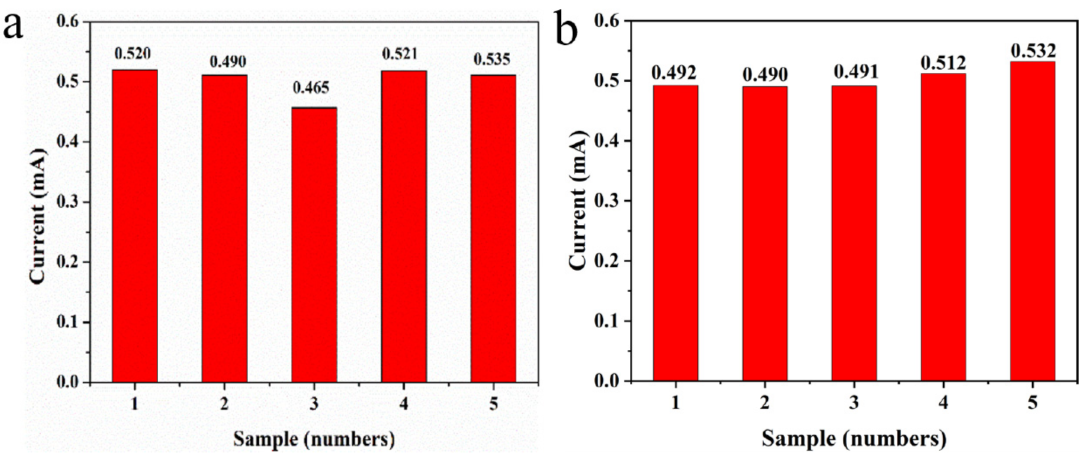

Figure S4. (**a**) The reproducibility of Cu/RTEG-F electrode, (**b**) The repeatability of Cu/RTEG-F electrode.

References

1 Anand, V. K.; Bukke, A.; Bhatt, K.; Kumar, S.; Sharma, S.; Goyal, R.; Virdi, G. S., Highly sensitive and reusable Cu+2/polyaniline/reduced graphene oxide nanocomposite ink-based non-enzymatic glucose sensor. *Appl. Phys.* A, **2020**, 126, 500.

2. Zhang, L.; Ye, C.; Li, X.; Ding, Y.; Liang, H.; Zhao, G.; Wang, Y., A CuNi/C Nanosheet Array Based on a Metal-Organic Framework Derivate as a Supersensitive Non-Enzymatic Glucose Sensor. *Nanomicro Lett.* **2018**, 10, 28.

3. Zhang, L.; Ding, Y.; Li, R.; Ye, C.; Zhao, G.; Wang, Y., Electrodeposition of ultra-long copper nanowires on a titanium foil electrode for nonenzymatic voltammetric sensing of glucose. *Microchim. Acta*. **2017**, 184, 2837–2843.

4. Wang, B.; Wu, Y.; Chen, Y.; Weng, B.; Li, C., Flexible paper sensor fabricated via in situ growth of Cu nanoflower on RGO sheets towards amperometrically non-enzymatic detection of glucose. *Sens. Actuators B: Chem*. **2017**, 238, 802–808.
